# Supplementary material for: Content-rich biological network constructed by mining PubMed abstracts
Source: BMC Bioinformatics. 2004 Oct 8;5:147. doi: 10.1186/1471-2105-5-147 (PMC528731; doi:10.1186/1471-2105-5-147)
Supplement: Additional File 2 — The original results of the above study (non-essential files are deleted to keep the file size under the limit set by BMC bioinformatics). [file 1471-2105-5-147-S2.bz2 › chilibotAdditionalFile2/dip10/230ID7931156E749/html/UL5_UL8.html]

 


 **UL5** and **UL8** 
  
Found 42 abstracts in PubMed, retrieved 10.  
 

 What does Google say? 
 PDF only 
| .edu only 

---

**Interactive relationship** (e.g. stimulation, inhibition, etc)

**Neutral relationship**- The interactions of the UL52 protein with the  **UL8**  and  **UL5**  proteins were analysed by using the yeast two hybrid system.  Ref: 10501495 J Gen Virol, 1999
- Inhibition of herpes simplex virus replication by a 2 amino thiazole via interactions with the helicase component of the  **UL5**   **UL8**  UL52 complex.  Ref: 9696789 J Virol, 1998
- On the other hand, the mutant protein retains its ability to interact with  **UL5**  as indicated by copurification and with  **UL8**  as indicated by a supershifted band in the gel mobility shift assay.  Ref: 10075707 J Biol Chem, 1999

**Non-interactive relationship** (e.g. studied together, co-existance, homology, etc.)

- Herpes simplex virus type 1 expresses a heterotrimeric helicase primase, the subunits of which are encoded by the viral  **UL5** ,  **UL8**  and UL52 genes.  Ref: 10501495 J Gen Virol, 1999
- The results shown in this paper are consistent with the model that the five proteins, ICP8,  **UL5** ,  **UL8** , UL9, and UL52, form a scaffold.  Ref: 12634381 J Virol, 2003
- The primase activity of the intertypic complex was 10 fold lower than that of HSV 1  **UL5**  HSV 1  **UL8**  HSV 1 UL52.  Ref: 9445019 J Virol, 1998
- The herpes simplex virus type 1  **UL5** ,  **UL8** , and UL52 genes encode an essential heterotrimeric DNA helicase primase that is responsible for concomitant DNA unwinding and primer synthesis at the viral DNA replication fork.  Ref: 9593724 J Biol Chem, 1998
- The ordered assembly of the herpes simplex virus HSV type 1 replication apparatus leading to replication compartments likely involves the initial assembly of five viral replication proteins, ICP8, UL9, and the heterotrimeric helicase primase complex  **UL5**   **UL8**  UL52, into replication foci.  Ref: 12634381 J Virol, 2003
- All three subunits of this complex  **UL5** ,  **UL8** , and UL52 are required for viral DNA replication in all cell types.  Ref: 9445019 J Virol, 1998
- We show that the HSV 1  **UL5** ,  **UL8** , UL29, UL30, UL42, and UL52 gene products along with the AAV Rep68 protein are sufficient to initiate replication on duplex DNA containing the AAV origins of replication, resulting in products several hundred nucleotides in length.  Ref: 11581393 J Virol, 2001
- Herpes simplex virus type 1 encodes a heterotrimeric helicase primase complex composed of the products of the  **UL5** , UL52, and  **UL8**  genes.  Ref: 11278618 J Biol Chem, 2001
- The UL6 locus of pseudorabies virus PRV was analyzed to reveal a gene cluster with homology to herpes simplex virus  **UL5** , UL6, UL7 and  **UL8** , Epstein Barr virus BBRF1 and BBRF2, and Kaposi sarcoma associated herpes virus ORF43 and ORF42.  Ref: 9458304 Oncol Rep< MedlineTA>Oncol Rep, 2001
- The intertypic complex HSV 2  **UL5**  HSV 1  **UL8**  HSV 1 UL52 was purified and biochemically characterized.  Ref: 9445019 J Virol, 1998
- Herpes simplex virus type 1 encodes a heterotrimeric helicase primase complex that is composed of the products of the  **UL5** , UL52, and  **UL8**  genes.  Ref: 10075707 J Biol Chem, 1999
- the heterotrimeric helicase primase complex, encoded by  **UL5** ,  **UL8** , and UL52 genes, is believed to unwind duplex viral DNA at replication forks.  Ref: 11642367 Prog Nucleic Acid Res Mol Biol, 2001
- T157602 inhibited reversibly the helicase activity of the HSV  **UL5**   **UL8**  UL52  **UL5**  8 52 helicase primase complex with an IC50 concentration of compound that yields 50% inhibition of 5 microM.  Ref: 9696789 J Virol, 1998
